# Supplementary material for: Cancer patients’ experiences of the diagnosis and treatment of incidental pulmonary embolism (a qualitative study)
Source: PLoS One. 2022 Oct 25;17(10):e0276754. doi: 10.1371/journal.pone.0276754 (PMC9595511; doi:10.1371/journal.pone.0276754)
Supplement: Attachment — Submitted filename: Responce to the reviewers.docx [file pone.0276754.s001.docx]

Dear Vanessa Carels,

Thank you for the opportunity to respond to the editor comments. We have addressed them point by point as described below. We think this has improved the quality of the review paper.

When submitting your revision, we need you to address these additional requirements.

1. Please ensure that your manuscript meets PLOS ONE's style requirements, including those for file naming. The PLOS ONE style templates can be found at

https://journals.plos.org/plosone/s/file?id=wjVg/PLOSOne_formatting_sample_main_body.pdf and

https://journals.plos.org/plosone/s/file?id=ba62/PLOSOne_formatting_sample_title_authors_affiliations.pdf".

Thank you. We have checked all the requirements and made the amended the files names.

2. Please review your reference list to ensure that it is complete and correct. If you have cited papers that have been retracted, please include the rationale for doing so in the manuscript text, or remove these references and replace them with relevant current references. Any changes to the reference list should be mentioned in the rebuttal letter that accompanies your revised manuscript. If you need to cite a retracted article, indicate the article’s retracted status in the References list and also include a citation and full reference for the retraction notice.

Thank you. We have checked the reference list and made the necessary amendments.

Reviewer #1: An interesting piece of qualitative research, which adds to the current body of literature. The article is well structured, appropriate methodology, outcomes supported by appropriate quotes. On the whole the article is easy to read and follow. I do however have a few suggestions questions which I hope once addressed will make the article easier to follow.

Thank you.

- Paragraph below (page 8) does not read well, difficult to make sense of it.

137 Although the diagnosis of pulmonary embolism was an incidental finding discovered on a CT scan ordered for cancer staging or follow up, participants reported having thrombosis (PE/DVT) related symptoms even though these had not been recognised. where carers were present and willing to participate, dyad interviews were conducted.

Thank you for pointing out this omission. We have amended this omission.

- Re quote below – this may be correct but need to check did the respondent say ‘wrap gloves’

198 “Just need to be careful, if I am going anywhere, and I wrap gloves if I work in the garden.”

Thank you for pointing out this omission. This now has been amended.

- What information do you present to support this statement below - in capturing information on the 11 patients interviewed did you ask about symptoms of PE? To enable you to state those without symptoms seemed to fare better?

260 Those without symptoms seemed to fare better psychologically, assuming “asymptomatic” to be “less dangerous”.

Thank you for raising this question. Patients were asked about their life with cancer before the diagnosis of IPE, which includes any new or worsening symptoms. We have this information mentioned in the analysis, Theme one: 137-141 “highlighted”. As well in Theme two: 175, 176 “highlighted”.

Thank you.
