# Supplementary material for: Cancer patients’ experiences of the diagnosis and treatment of incidental pulmonary embolism (a qualitative study)
Source: PLoS One. 2022 Oct 25;17(10):e0276754. doi: 10.1371/journal.pone.0276754 (PMC9595511; doi:10.1371/journal.pone.0276754)
Supplement: Attachment — Submitted filename: Responce to the revieweres.docx [file pone.0276754.s002.docx]

Dear Yoshihiro Fukumoto

Thank you for the opportunity to respond to the editor comments. We have addressed them point by point as described below. We think this has improved the quality of the review paper.

Reviewer #2: This study was a qualitative research regarding with cancer patients’ experience of the diagnosis and treatment of IPE. Although this study addresses interesting issue, there are some drawbacks in the manuscript as described below;

1. This study was a single center study with a small number of patients. How have the authors estimate the required number of subjects for this qualitative research?

Thank you for raising this question.

This was a convenience sample of consenting participants completing an observational survey study. (Line 78/79 highlighted). We adopt the theoretical saturation concept in this qualitative study (the point at which the data collection process no longer offers any new or relevant data) (line 95 highlighted and referenced). This is because qualitative research methods are often concerned with an in-depth understanding of a phenomenon or are focused on meaning (and heterogeneities in meaning)—which are often centred on the how and why of a particular issue, process, situation, subculture, scene or set of social interactions.

2. Why did the authors choose qualitative study to explore cancer patients’ experience of the diagnosis and treatment of IPE? Please mention the reason it was difficult to explore this issue by quantitative research.

Thank you for raising this question.

The objective of this study was to explore cancer patients’ experience of the diagnosis of and living with incidental pulmonary embolism treated with anticoagulation. (Line 20/21 highlighted) and statistical procedures and numeric data were insufficient to capture how patients feel about their diagnosis and its effects on their daily life.

The aim of this qualitative research is to understand the social reality of individuals cultures as nearly as possible as its participants feel it or live it, rather than make statistical inferences. This involves asking participants about their experiences of things that happen in their lives. It enables researchers to obtain insights into what it feels like to be another person and to understand the world as another experience it, whereas quantitative research may not provide definitive answers to such complex questions.

Reviewer #3: First of all, the current new Reviewer (for revision) would like to congratulate the authors for the current interesting report. The authors seemed to revise the manuscript appropriately according to the previous initial review. The Reviewer do not have a major comment and would like to raise a few minor comments for hope to be helpful for the authors before publication.

Thank you.

Minor comments)

Title: The current study was “a qualitative study”, not a quantitative study. For general readers, it could be helpful to describe “a qualitative study” in the title.

Thank you for pointing out this omission. We have amended this omission. (Title highlighted)

Results: In the study period, how many potential patients were there? (All IPE patients) 13? If possible, please add the information, because this information could show readers the absence or presence of selection bias of patients.

Thank you for raising this question.

Yes there were 13 potential patients. All been approached to participate. Two declined without giving any reason. (Line 79/80 highlighted).

In Conclusions section, the authors described that “Routine education for patients and clinicians should be provided.” However, as a clear message for general readers, routine education seemed to be vague description. What did the authors would like to intend?

Thank you for raising this question.

In the implications section of the paper, we referred to some potential ways that can be used to implement patients and health care education in patients’ care process, in the same way as patients are educated about neutropenic sepsis and spinal cord compression. (Line 312-317 highlighted).

However, we did not specify any potential actions of education as this need to be proved by future research, and this was out of the scope of this study.
